# Efficacy and Accuracy of Using Magnetic Seed for Preoperative Non-Palpable Breast Lesions Localization: Our Experience with Magseed

**DOI:** 10.3390/curroncol29110667

**Published:** 2022-11-07

**Authors:** Anna D’Angelo, Charlotte Marguerite Lucille Trombadori, Flavia Caprini, Stefano Lo Cicero, Valentina Longo, Francesca Ferrara, Simone Palma, Marco Conti, Antonio Franco, Lorenzo Scardina, Sabatino D’Archi, Paolo Belli, Riccardo Manfredi

**Affiliations:** 1 Dipartimento di Diagnostica per Immagini, Radioterapia Oncologica ed Ematologia, Fondazione Policlinico Universitario “A. Gemelli”, IRCCS, 00168 Rome, Italy; 2 Breast Unit, Department of Women, Children and Public Health Sciences, Fondazione Policlinico Universitario Agostino Gemelli IRCCS, 00168 Rome, Italy

**Keywords:** Magseed, breast cancer, preoperative localization, magnetic seed

## Abstract

In this retrospective study we share our single-center experience using a magnetic seed for the preoperative localization of non-palpable breast lesions. Patients who underwent a preoperative localization with Magseed^®^ (Endomagnetics, Cambridge, UK) placement between 2020 and 2022 were enrolled. Indications to Magseed placement have been established during multidisciplinary meetings prior to surgery and all patients underwent breast-conserving surgery (BCS). 45 patients were included. Magnetic seeds have been introduced under ultrasound guidance in 40 patients (88.9%) and under stereotactic guidance in 5 patients (11.1%). We registered a highly successful placement rate (97.8%), with only one case of migration (2.2%). After BCS, all the magnetic seeds were recovered (100% retrieval rate). The re-excision rate for positive margins was 0%. Our experience, with a highly successful placement and retrieval rate and a re-excision rate equal to 0%, is consistent with the encouraging literature published on Magseed so far, suggesting this technique to be extremely effective. Moreover, our single case of seed migration supports the existing data stating that Magseed migration is rare. In conclusion, despite acknowledging Magseed limitations, we highly value the advantages linked to this technique, and we, therefore, uphold its use.

## 1. Introduction

Breast cancer (BC) has become the leading cause of global cancer incidence and the fifth leading cause of cancer mortality worldwide [1]. Considering the increasing of BC incidence [1], principally related to improvements in diagnostic techniques and the aging of the population, the detection of non-palpable breast lesions has become increasingly frequent.

For non-palpable BC, the treatment of choice is Breast Conserving Surgery (BCS) [2]. In order to be successful in achieving a complete excision of the lesion, a correct pre-operative localization is required. Thus, accurate and state-of-the-art localization is a pivotal step in the management of a BC patient, with an increasing demand for the development of reliable localization approaches for non-palpable lesions.

Recently, localization techniques have undergone constant improvements. One of the first types of localization technique was the wire guide localization (WGL), still widely used, consisting of locating a wire inside the lesion under ultrasound or mammography guidance. The main limitations of this procedure are the need to perform it on the same day of the surgery, the risk of displacement, and a worse aesthetic result as the breast tissue along the path of the thread must be removed. 

Given the above strains, non-wire localization systems have been developed. One of the earliest non-wire systems to be implemented was the Radio-guided Occult Lesion Localization (ROLL) [3] which uses a radioactive marker. However, this localization method requires to be performed on the day of the surgery or a few days before, depending on the half-life of the radioactive molecule. Furthermore, this technique necessitates a nuclear medicine service within the hospital and determines a risk of exposure for both the operator and the patient. Therefore, over the following years, non-wire and non-radioactive localization tools have been implemented, such as the Radio-Frequency Identification tag (RFID), the Savi-Scout and the magnetic seed systems. 

The RFID system uses radio frequencies and, despite some limitations [4], is considered safe and effective for non-palpable breast lesions localization, with re-excision rates similar to WGL. It can be deployed inside the lesions the day before surgery. The Savi-Scout system is another non-wire and non-radioactive alternative technique; it uses a micro-impulse infrared radar to localize the lesions and is particularly useful for patients that need MRI examinations during follow-up, without artifacts [5].

Magnetic localization techniques were developed as an alternative to the methods mentioned above [6]. The Magseed^®^ (Endomagnetics, Cambridge, UK), a non-wire and non-radioactive paramagnetic localization tool was approved in 2016 by the Food and Drug Administration (FDA) [7]. It has the crucial advantage that it can be introduced inside the lesion and can remain in place until the time of BCS, despite the first indications that recommend the placement of Magseed up to 30 days before surgery [8]. Hence, the workload of the radiologist before the surgery is reduced as well as delays in surgical theaters due to localization procedures. 

**Figure 1:**
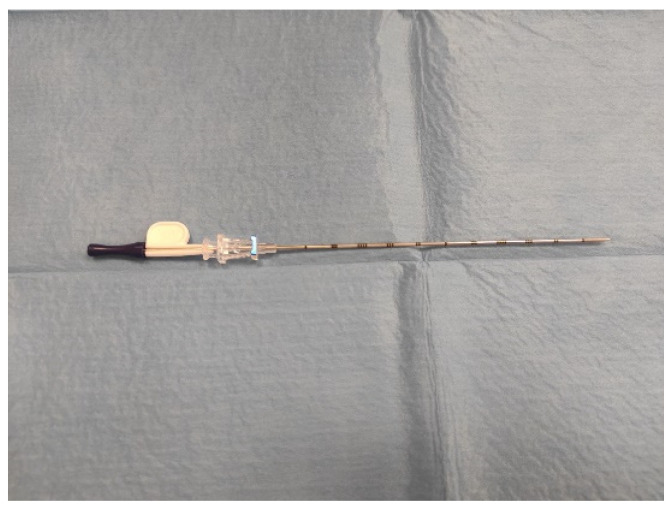
The 18-gauge Magseed introducer.

Magseed is composed of a seed of 5 × 1 mm inserted within an 18-G sterile needle (Figure 1), and its introduction can be performed either under ultrasound or mammography guidance. Following accurate disinfection of the skin and the injection of local anesthesia, the needle with the magnetic marker is inserted and centered with its distal end as proximal as possible to the target lesion, where the marker is released. A double-view mammography is performed to assess the right placement of the seed. On the day of the surgery, an ultrasound or a mammography examination is performed to evaluate the correct position of the marker, to avoid migration. The magnetic clip is then identified during surgery by the SentiMag^®^ (Endomagnetics, Inc., Cambridge, UK) probe, which generates a magnetic field and magnetizes the seed. During surgery, the distance of the probe from the Magseed is indicated by numerical values displayed on the monitor and with audio feedback. The magnetic seed is considered detectable within a distance that is around 4 cm away from the SentiMag [9]. 

This retrospective study aimed to share our experience with magnetic seed and to evaluate its efficacy and accuracy for preoperative non-palpable breast lesion localizations.

## 2. Materials and Methods

### 2.1. Patients

Our institutional review board approved this single-institution retrospective study. A total of 45 patients who underwent Magseed placement between June 2020 and February 2022 were included. Inclusion criteria were: 18 years old or older, single non-palpable breast lesion and surgery performed in our center. Exclusion criteria were: palpable breast lesions and patients who underwent previous chemotherapy treatment. Each patient enrolled signed informed consent before undergoing the interventional procedure.

### 2.2. Procedure

**Figure 2:**
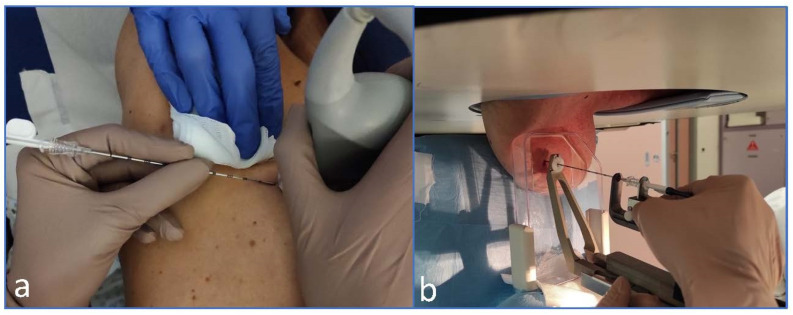
Magseed insertion: ultrasound (**a**) and stereotactic guidance (**b**).

**Figure 3:**
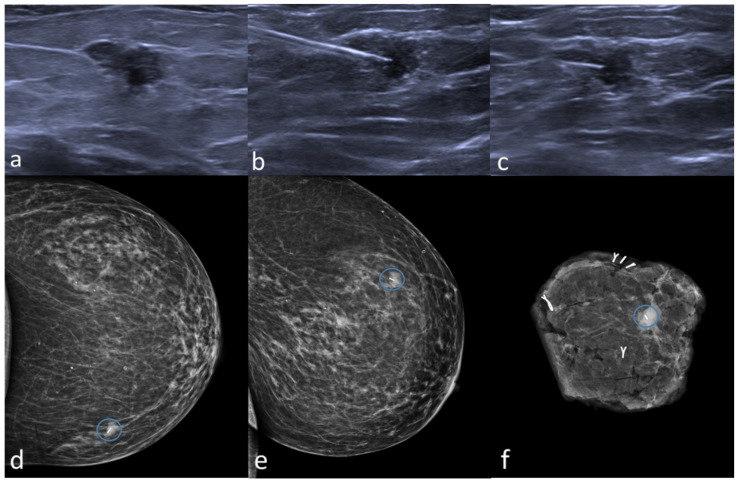
Magseed placement under ultrasound guidance. Preoperative ultrasound localization of a non-palpable, hypoechoic lesion (invasive ductal carcinoma) in the left upper inner quadrant (**a**), and placement of Magseed inside the lesion (**b**,**c**). Preoperative mammogram in two views confirms the correct placement of the Magseed (blue circle, (**d**,**e**)). The surgical specimen shows the presence of both the tumor and Magseed (blue circle, (**f**)).

The placement of Magseed was decided and approved by a multidisciplinary meeting between breast surgeons, plastic surgeons and radiologists. A total of 45 Magseeds were placed, 40 under ultrasound guidance (88.9%) and 5 under stereotactic placement (11.1%) (Figure 2). Each procedure followed accurate disinfection of the skin (chlorhexidine) and the injection of local anesthetic (Mepicain 2%); after the introduction of the magnetic seed, an ultrasound and a mammogram (two-views mammography, mediolateral oblique, and craniocaudal views) were performed, in order to document the correct position of the marker (Figure 3). On the day of the surgery, a double-view mammography is performed to verify the correct position of the seed (Figure 3). 

**Figure 4:**
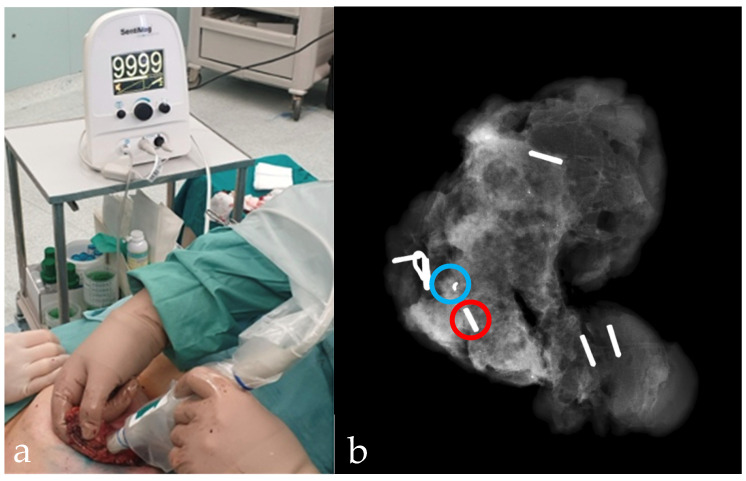
The SentiMag probe (**a**), used intraoperatively to locate the Magseed using a small and transient magnetic field that magnetizes the clip making it recognizable to the probe itself. In (**b**) radiographic examination of the surgical specimen shows the Magseed (red circle) adjacent to the previously clipped lesion (blue circle).

The Magseed was identified during surgery using the SentiMag probe (Figure 4); the audio signal has a frequency that varies according to the intensity of the magnetic field or with the distance of the seed from the probe, helping the surgeon to find the lesion. At the end of the surgery, surgical specimen radiography in craniocaudal view with the tomosynthesis (the routine practice in our center) was performed to assess the presence of the Magseed and to evaluate the distance between the lesion and the close margins (Figure 3f and Figure 4b). If the lesion is detected on the surgical specimen margin at the radiography, intraoperative widening is performed. After that, the surgical specimen was examined by the pathologist for the histological assessment and for the evaluation of margin status (“no ink on tumor”) [10]. 

We evaluated patient demographics, lesions characteristics, Magseed localization features (ultrasound-guided or stereotactic-guided), seed migration, successful Magseed detection and retrieval in the surgical specimen, time of Magseed placement (in minutes) and time between Magseed placement and surgery (in days).

## 3. Results

A total of 45 patients were included in the study. The mean patient age was 57, 58 years (range 31–80). The preoperative mean size of the breast lesions was 8.8 millimeters (mm) (range 3–18 mm) (Table 1). The patients enrolled in the study underwent BCS. The intraoperative widening and the re-excision rate for positive margins were 0% (Table 1).

The pathological examination found a prevalence of malignant lesions, of which 77.8% were invasive ductal carcinoma (IDC) and 8.8% ductal carcinoma in situ (DCIS), while the remaining 13.4% were B3 lesions [11] (Table 1).

**Figure 5:**
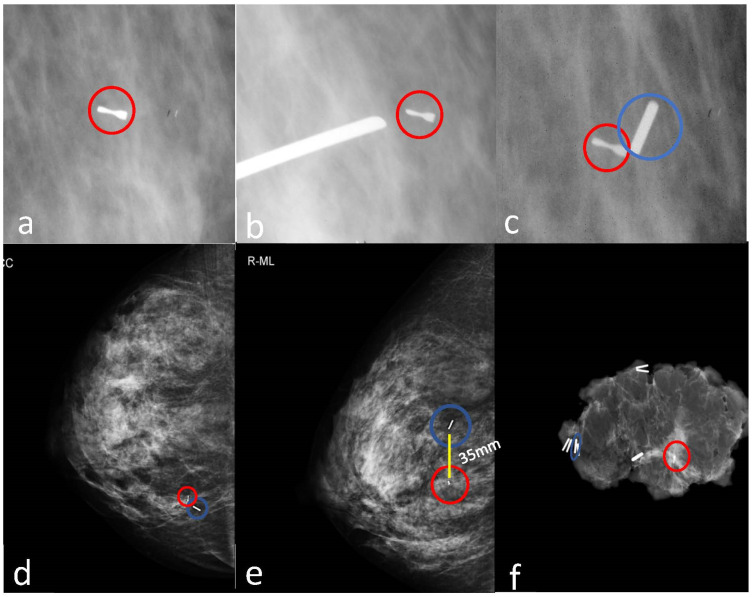
Misplaced Magseed. In (**a**–**c**), the placement of the Magseed (blue circle in **c**) under stereotactic guidance in the site of a previous stereotactic biopsy with a metallic clip (the red circles). Preoperative mammography in two views (**d**,**e**) shows a cranial displacement of the Magseed (blue circles in (**e**)) at a distance of 35 millimeters (mm) from the biopsy-clipped lesion (red circles). The surgical specimen radiogram reveals the Magseed (blue circle) and the clipped lesion (red circle) (**f**) correctly removed.

Magseed localization features were reported in Table 2. A total of 40 magnetic seeds were placed under ultrasound guidance (88.9%) and 5 under stereotactic guidance (11.1%) (Table 2). No immediate complications after placement were observed (0.0%) and we obtained a high placement success rate (97.8%) since all markers were correctly positioned, except for one case of migration of the marker placed under stereotactic guidance (2.2%) (Figure 5). All magnetic seeds were recovered in the surgical specimens (100%) (Table 2). 

Most of the seeds were placed some days before surgery or the same day of surgery (average time was 3.46 days). On average, Magseed placement took about 5.5 min. 

## 4. Discussion

Magseed represents one of the most promising options for the localization of non-palpable breast lesions. Recent data on its use are unarguably encouraging, proving this technique to be extremely effective.

A recent systematic review by Gera et al. [9] demonstrated the effectiveness of the Magseed in localizing non-palpable breast lesions, particularly as compared to the WGL. Indeed, the results obtained from the analysis of 16 studies, with a highly successful localization and retrieval rate (99.86%) and a relatively low re-excision rate (11.25%), support the use of this technique. In a multicenter clinical retrospective trial, Žateckýa et al. [12] evaluated a pilot use of the magnetic seed in 34 breast tumors. They reported negative margins after surgery in 29 out of 34 (85.3%) patients. Positive resection margins were found in 4 out of 34 patients (11.8%), and 1 case of seed migration was reported, with a rate of 14.7% (5/34) re-excision rate. Several studies reported no seed migration after perioperative tumor marking [13,14], being this a rare occurrence. The experience of our center is consistent with previous literature with a highly successful localization rate (97.8%); conversely, compared to other studies, our intraoperative widening and re-excision rate are lower (0%), with 0% of positive margins.

As we said above, one of the benefits of the Magseed is the possibility to deploy the seed several days ahead of surgery, enabling a more efficient and flexible organization of the workflow [15]. For this reason, the magnetic seed could be useful in case of a long follow-up, especially during neoadjuvant chemotherapy, despite the well-known signal-void MRI artifact [7]. This limitation significantly affects image quality and can, in some cases, hamper the use of the MRI for the follow-up after therapy. However, contrast-enhanced mammography can be chosen as a good and performing alternative for those patients with wider artifacts. We reported a mean time of 3.46 days between seed deployment and the day of surgery, even if most of the localizations took place on the same day of the surgery. We reported one case (2.2%) of seed migration, which is in line with the literature [14]. 

One limitation of the magnetic seed is related to the depth of the lesion in the breast, measured as its distance from the skin. As we stated in the introduction, Magseed is considered detectable within a distance of around 4 cm away from the SentiMag, making it harder for deeper lesions to be found [8,15]. However, many studies in literature report that intraoperatively using palpation with the detector, seeds far deeper were detected [9,15]. Given this, it may be preferable for extremely deep lesions to use WGL. Another important aspect that disincentives the use of Magseed is the cost [16]. In our analysis, we have not considered either. 

This study has some limitations. The experience was limited to one center, and above all, it included a small cohort of patients. Moreover, we did not analyze some variables (e.g., depth from the skin of the breast lesion and Magseed costs), and the time between Magseed placement and surgery was short (3.46 days), probably affecting the data regarding the seed migration. 

## 5. Conclusions

With the limits mentioned above, our single-center experience is consistent with the data reported in literature, suggesting this technique is effective in the preoperative localization of non-palpable breast lesions. 

Future studies including a bigger sample size and a longer time interval between seed placement and surgery are needed to validate our results. 

## Figures and Tables

**Table 1:** Clinical and surgical data. Millimeters (mm). Ductal carcinoma in situ (DCIS). Invasive ductal carcinoma (IDC). B3 lesions according to the lexicon BI-RADS^®^.

Patients Age (Years)	57.58 (range 31–80)
Breast lesions dimension (mm)	8.8 (range 3–18)
Type of surgery:	
Lumpectomy	3
Quadrantectomy	43
Re-excision rate	0%
Intraoperative widening	0%
Post-operative histology:	
DCIS	4 (8.8%)
IDC	35 (77.8%)
B3	6 (13.4%)

**Table 2:** Magseed localization data. Days (d). Minutes (min).

Total Magseed Placed	45
Localization modality:	
Ultrasound localization	40 (88.9%)
Stereotactic localization	5 (11.1 %)
Seed migration/malpositioning	1 (2.2%)
Successful detection and retrieval	45 (100%)
Time between Magseed placement and surgery (d)	3.46
Time for Magseed placement (min)	5.5

## Data Availability

Not applicable.
